# Data Acquisition and Processing in Biology and Medicine

**DOI:** 10.1155/2015/538031

**Published:** 2015-08-26

**Authors:** Cheng-Hong Yang, Yu-Jie Huang, An Liu, Yi Rong, Tsair-Fwu Lee

**Affiliations:** ^1^Medical Physics & Informatics Laboratory of Electronics Engineering, National Kaohsiung University of Applied Sciences, Kaohsiung 80778, Taiwan; ^2^Graduate Institute of Clinical Medicine, Kaohsiung Medical University, Kaohsiung 80708, Taiwan; ^3^Department of Radiation Oncology, Kaohsiung Chang Gung Memorial Hospital and Chang Gung University College of Medicine, Kaohsiung 83305, Taiwan; ^4^Department of Radiation Oncology, City of Hope National Medical Center, Duarte, CA 91010, USA; ^5^Department of Radiation Oncology, The Ohio State University, Columbus, OH 43210, USA

There are huge amounts of information in the real world. These data can be acquired and processed for useful applications. Data acquisition is the process of sampling signals that measures real world physical conditions and converts the resulting samples into digital numeric values that can be manipulated by computer. Data processing is, broadly, “the collection and manipulation of data to produce meaningful information” [1, 2].

Conventionally, the data acquisition and processing are the usual studying method in engineering. There are also data that could be caught and manipulated from biological objects, either human or animal. However, the data in biology and medicine are not as direct and significant as physical signals. With the development of technology, the data could be caught by sensors from biological objects directly or indirectly. The data then could be processed to useful information for analysis, diagnosis, and treatment. Computer tomography is the best example [3]. The X-ray passes through human body and is detected by photoelectric sensor; the computer reconstructs the axial images according to the data from sensors. It illustrates the delicate anatomic structure of human body without incision. This technology has a great benefit to human health. Therefore, through advancements in data acquisition and processing in biology and medicine, the human health could have a great advantage and improvement.

The main focus of this special issue is on the advance of the data acquisition and processing techniques to facilitate the development in biology and medicine. The special issue explores and summarizes the most recent progressing of the data acquisition and processing in biology and medicine. We have accepted 5 papers after peer review. Two of them are about the data processing of electroencephalogram (EEG) to facilitate diagnosis [4]. C. Zhang et al. described the automatic artifact removal from EEG data based on a priori artifact information. In their study, a priori artifact information acquired online was introduced into wavelet-independent component analysis (WICA) to realize automatic artifact removal for variable subjects and EEG acquisition environments. Their results showed that the method significantly improved the classification accuracies for motor imagery and emotion recognition. Y.-S. Choi reported information-theoretical quantifier of brain rhythm based on data-driven multiscale representation for neurological deficit evaluation in rat. They presented a novel multiscale Renyi entropy framework for analysis of EEG signals. Analysis of experimental EEG recording has shown that multiscale Renyi entropy correlates well with clinically relevant measures of neurological deficits. This study lays the foundation for applying this novel approach to clinical studies of human EEG signals recorded during comparable episodes of brain injury resulting from global ischemia after cardiac arrest as well as other clinical situations such as traumatic brain injury.

Besides EEG, electromyography (EMG) could also be processed for diagnosis [5]. T.-F. Lee et al. analyzed EMG equivalent uniform voltage response to diagnose tennis elbow. EMG is also transmission signal that could be acquired and analyzed to help diagnosis in muscular system. The features of surface EMG in lateral epicondylitis patients can be extracted. The prediction model can be formed using logistic and probit techniques to diagnose tennis elbow. EMG analysis also has potential to detect medical abnormalities, activation level, or recruitment order or to analyze the biomechanics of human or animal movement. R. Peña et al. reported a clinically practical technique to embed physiological signals in video for medicine healthcare. Their technique successfully embedded samples of six EEG signals into encoded video sequences with high, medium, and low motion. Their technique also extracted the hidden samples from the encoded video sequences without loss of information. It is an important aid to improve the safety and effectiveness in medical care.

C.-H. Yang et al. used a particle swarm optimization (PSO) algorithm to identify the possible protective models of breast cancer association in terms of the single nucleotide polymorphisms (SNPs) of ORAI calcium release-activated calcium modulator 1 (ORAI1) gene based on a published data set of 345 female breast cancer patients and 290 female controls. Two SNPs (rs12320939 and rs12313273) were found to be the most essential components to protectively associate in breast cancer. PSO identified SNP model may enhance the detection of genetic variants to disease or cancer susceptibility. Therefore, their findings provided the important information regarding combinational patterns of SNPs located in the relevant genes [6, 7].

The advancement of the data acquisition and processing techniques should be the key to facilitate the development in biology and medicine. The potential topics include, but are not limited to, physiological data acquisition and processing; the development of biosensor; image acquisition and processing in biology and medicine; computer in biology and medicine, hardware setup, and software development; database acquisition and analysis in biology and medicine; and computing algorithm and optimization in biology and medicine. The goal and focus of this special issue are the advance of the data acquisition and processing techniques to facilitate the development in biology and medicine. The technological advancements in this decade have been immense, especially in sensors and computers. There is tremendous amount of technological advancement that could be used in biology and medicine. More technical breakthroughs in data acquisition and processing will be developed in the future, which may present the chance and challenge for researchers in this field.

*Cheng-Hong Yang* *Yu-Jie Huang* *An Liu* *Yi Rong* *Tsair-Fwu Lee*
